# Large anomalous Nernst effect in a skyrmion crystal

**DOI:** 10.1038/srep28076

**Published:** 2016-06-16

**Authors:** Yo Pierre Mizuta, Fumiyuki Ishii

**Affiliations:** 1 Graduate School of Natural Science and Technology, Kanazawa University, Kanazawa, 920-1192 Japan; 2 Faculty of Mathematics and Physics, Kanazawa University, Kanazawa, 920-1192 Japan

## Abstract

Thermoelectric properties of a model skyrmion crystal were theoretically investigated, and it was found that its large anomalous Hall conductivity, corresponding to large Chern numbers induced by its peculiar spin structure leads to a large transverse thermoelectric voltage through the anomalous Nernst effect. This implies the possibility of finding good thermoelectric materials among skyrmion systems, and thus motivates our quests for them by means of the first-principles calculations as were employed in this study.

Thermoelectric (TE) generation, harvesting waste heat and turning it into electricity, should play an important role in realizing more energy-efficient society and overcoming the global warming. Nevertheless, it is yet to be widely used, mainly due to its still limited efficiency. There have been many studies pursuing highly efficient TE systems with a large value of figure of merit: *Z*_*X*_ = *σX*^2^/*κ*, where *σ* and *κ* are longitudinal electrical and thermal conductivity respectively, and *X* = *S* or *N* is the Seebeck or Nernst coefficient depending on whether we use the longitudinal or transverse voltage for power generation. Here we omitted labels *xx* or *yy* in *σ* and *κ* by assuming an isotropic system. Among those, our study sheds light on the anomalous effect of electrical conduction perpendicular to an electric field (anomalous Hall effect, AHE)^1^ or to a temperature gradient (anomalous Nernst effect, ANE)^2^ on TE performance, focusing on a particular contribution to the conductivity of AHE (ANE), namely the so-called *intrinsic* term 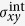 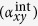 expressed as a functional of Berry curvature^3^ Ω(**k**) ≡ *i*〈∂_**k**_*u*|×|∂_**k**_*u*〉 in momentum (**k**) space as in the second formula of Eq. (2), where |*u*_**k**_〉 is the periodic part of a Bloch state.

Systems hosting AHE/ANE are found in magnetic materials, both normal semiconductors^4^ and topological insulators^5^. We previously studied simple models of 2D electron gas in an interface composed of those materials^6^,^7^. The anomalous effect on Seebeck coefficient was fairly large there, but remained rather small compared to what will be reported in this paper, which can mainly be attributed to the limited magnitude of the anomalous Hall conductivity (AHC) 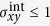 there [The unit is taken to be *e*^2^/*h* (with *e* and *h* being the electron charge and the Plank’s constant respectively) for the AHC in 2D in this paper].

There have been several ideas proposed for obtaining large AHC, such as the manipulation of massive Dirac cones (each of them being the source of 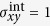) by controlling parameters^8^, or the extension of system dimension in the normal-to-plane direction (2D to 3D)^9^.

What we focus here is another one, namely, the control of real-space spin textures which are well known to induce another contribution to the AHE/ANE, often called the *topological* Hall/Nernst conductivity^10^ 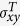 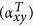 that also has a geometrical meaning related to 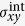 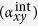. In terms of Berry-phase theory^3^, the emergence of *topological* terms in the continuum limit (spin variation scale 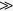 atomic spacing), is an analogue of the ordinary Hall effect with the external *B* field just replaced by *spin magnetic field B*_spin_, which is the real-space Berry curvature Ω(**R**) itself, proportional to a quantity called *spin scalar chirality χ*_*ijk*_ ≡ **m**_*i*_ · **m**_*j*_ × **m**_*k*_ reflecting the geometrical structure spanned by each spin trio (**m**_*i*_, **m**_*j*_, **m**_*k*_). Although it should be better to treat 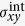 and 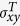 on an equal footing, we will omit the latter (*topological*) term in the main part of this paper because the validity of the simple relation *B*_spin_ ∝ Ω(**R**) is not clear for our system of rather short spin variation scale. Still, the omission can be justified within this approximation (see Part A of Supplemental Information for details).

Among many possible spin textures, what we target here is the so-called skyrmion crystal (SkX) phase observed even near room-temperature^11^, where skyrmions, particle-like spin whirls, align on a lattice. The skyrmion, originally discussed in nuclear physics, has been studied extensively these days in condensed matter physics as well. Typical SkX-hosting materials are some of the transition metal silicides/germanides: MnSi^12^, MnGe^13^, FeGe^11^, or heterostructures such as monolayer Fe on Ir(111)^14^, in all of which the Dzyaloshinskii-Moriya term, a spin-orbit coupling effect peculiar to their inversion-asymmetric crystal structure, plays a crucial role in the emergence of skyrmions.

Regarding the AHE in skyrmionic systems, quantum AHE (QAHE), i.e. the AHE with a quantized AHC 
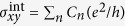
 with an integer *C*_*n*_ called Chern number of *n*-th band, has recently been predicted in some SkX models where the conduction electron spins are exchange-coupled to the SkX either strongly^15^ or weakly^16^, preceded by a report of QAHE in a meron (half-skyrmion) crystal^17^. In this paper we focus on the case Hamamoto *et al.* picked out^15^, because of its particularly large 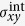 implying the possibility of large ANE as well, thanks to the close relation between AHE and ANE^2^,^18^, and that is the main point we confirmed in this study.

At the end of this section, we stress that the computational method used in our study is based on first-principles, which can be applied to the exploration of realistic materials of SkX etc. from the same perspective.

## Expressions of thermoelectric quantities

The formulae for the thermoelectric coefficients to be evaluated follow from the linear response relation of charge current: 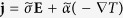, where **E** and 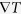 are the electric field and temperature gradient present in the sample.

Using the conductivity tensors 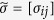 and 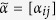, we obtain


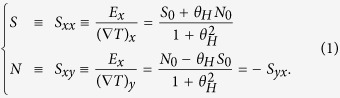


Here we defined *S*_0_ ≡ *α*_*xx*_/*σ*_*xx*_, *θ*_*H*_ ≡ *σ*_*xy*_/*σ*_*xx*_, *N*_0_ ≡ *α*_*xy*_/*σ*_*xx*_ for a simpler notation. The conductivities are expressed as^19^,^20^,^21^ 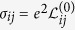, 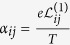, with 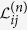 defined as 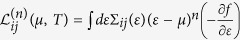, which in turn is a functional of the function Σ_*ij*_(*ε*) explicitly showing the energy dependence proper to the considered system, which is written as,


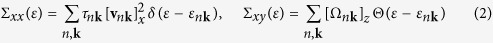


In the above formulae, *e*(<0), *τ*_*n***k**_, *f*(*ε*, *μ*), **v**_*n***k**_, *ε*_*n***k**_, Ω_*n***k**_, *μ*, and Θ stand for the electron’s charge, relaxation time (hereafter assumed to take a constant value *τ*), the Fermi-Dirac distribution function, (velocity, energy and **k**-space Berry curvature) of an electron with wave number **k**, chemical potential and Heaviside step function, respectively. The subscript index *n* is put on each band-resolved quantity.

Throughout this paper, since our 2D system has no periodicity in *z* direction, we discuss 
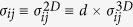
, which is independent of the film thickness *d* and has the dimension of *e*^2^/*h* whose unit can be (Ohm)^−1^, instead of *real* conductivity 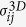.

Note that the conventionally-discussed Seebeck coefficient *S*_0_ estimated without considering Berry curvature is obtained by setting *θ*_*H*_ = 0 and *N*_0_ = 0 in Eq. (1).

See Part B of Supplemental Information for some mathematical expressions needed to obtain the above expressions.

## Model

**Figure 1:**
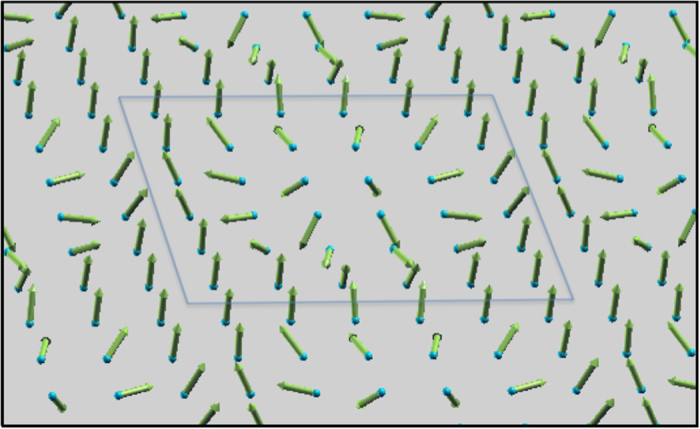
A view of an unit cell of 6 × 6 SkX.

We consider a magnetic SkX on a two dimensional square lattice, with its unit cell of lattice constant 2*λ* = 1.98 nm containing 6 × 6 spins, thus the atomic lattice spacing *a* = 2*λ*/6 being 3.3 Å. This size of 2*λ*  roughly corresponds to the smallest 3 nm as observed in MnGe^13^ (We studied larger ones as well, and the obtained size-dependence is shown in Supplemental Information C). The spin configuration is equivalent to the one Hamamoto *et al.* studied^15^, i.e., the spherical coordinates of spin **m**(**r**_*i*_) located at site *i* are set as *θ*_*i*_ = *π*(1 − *r*_*i*_/*λ*) for *r*_*i*_ < *λ* and *θ*_*i*_ = 0 for *r*_*i*_ > *λ*, along with *ϕ*_*i*_ = tan^−1^(*y*_*i*_/*x*_*i*_) + (arbitrary constant). The spin modulation in the system is shown in Fig. 1. This fixing of spins corresponds to the strong limit of Hund’s coupling to localized spins. In order to simulate the simplest case, we assume each spin is that of a hydrogen atom. We suppose our model, in the sense that it has a single orbital per site, is somewhat close to such *e*_*g*_-orbital systems as SrFeO_3_ films, where the emergence of SkX-like topological spin textures has been implied^22^. The model has several limitations, though, with regard to its coverage of experimental situations, which are described in Supplemental Information E.

## Methods

Our calculations consist of three steps: With *OpenMX* code^23^ (1) obtain the electronic Bloch states {|Ψ_*n***k**_〉 = *e*^*i***k**·**r**^|*u*_*n***k**_〉} and corresponding eigenenergies {*ε*_*n***k**_} of the target SkX, and using a functionality^24^ implemented in *OpenMX*, which is based on the formalism proposed by Marzari *et al.*^25^ and Souza *et al.*^26^, calculate their overlaps 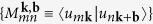 between neighboring **k**-points **k** and **k** + **b** on a grid, and the projections 
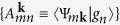
 of a guessed set of localized orbitals {|*g*_*n*_〉} onto the Bloch states, and with *Wannier90* code^27^ (2) construct maximally localized Wannier functions (MLWF) as particular linear combinations of {|*g*_*n*_〉} at each **k** point using the three sets of data passed from step(1): {*ε*_*n***k**_}, 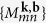 and 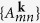, then finally (3) compute from the obtained MLWF all the necessary transport quantities {*σ*_*ij*_, *α*_*ij*_} [and finally Eq. (1)] expressed according to the Boltzmann semiclassical transport theory, where we adopted constant-relaxation-time approximation with a fixed value of *τ* = 0.1 ps, a value expected to be realistic^28^ (We will find, however, later in the discussions that our results are very sensitive to the choice of *τ*). In step(1), two *s*- and one *p*-character numerical pseudo-atomic orbitals with cutoff radius of 7 bohr was assigned to each H atom. The present calculation for a non-collinear magnetic system was realized by applying a spin-constraining method^29^ in the non-collinear density functional theory^30^. Step(1) yielded 6 × 6 = 36 non-spin-degenerate occupied bands and the equal number of unoccupied ones, among which only the former 36 bands were used in step(2) to construct MLWF and interpolated with them to calculate the conductivities. In step(3), two modules were used in *Wannier90*: *berry* module based on the formalism proposed by Wang *et al.*^31^ to compute Σ_*xy*_(*ε*) and *boltzwann* module introduced by Pizzi *et al.*^32^ to compute *σ*_*xx*_ and *S*_0_, in both of which the sampling for integrations was performed on 50 × 50 **k**-points. Besides these, numerical integrations were carried out to evaluate *σ*_*xy*_ and *α*_*xy*_ from Σ_*xy*_(*ε*).

The above procedure was tested in the following manner: For 4 × 4 SkX, the calculated band structure and the Fermi energy dependence of AHC were in overall agreement with the ones previously reported in the tight-binding model study^15^, confirming the reproducibility of the similar situation by different approaches.

In addition, the AHC and the Berry curvature were computed also via another formalism, which is advantageous in the sense that it can identify the Chern number assigned to each band (see Supplemantal Information D), and the consistency was confirmed between the results from the two different methods.

## Results and Discussions

### Electronic structure and conductivities

**Figure 2:**
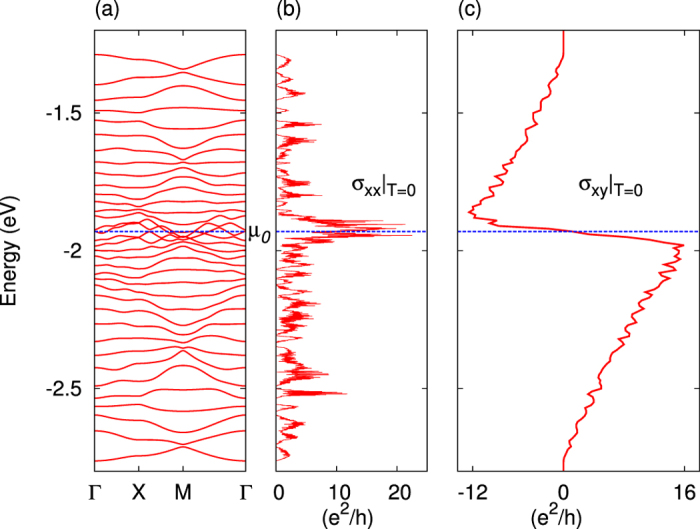
(**a**) Band structure and Fermi energy dependence of (**b**) longitudinal and (**c**) anomalous Hall conductivity of 6 × 6 SkX. The blue dashed line indicates the *μ*_0_ mentioned in the main text.

First we show the obtained band structure of the 36 occupied states in Fig. 2(a). We notice there that each band is well isolated from each other, except for the four bands around the middle energy range [−2.0, −1.9] eV, which we shall hereafter refer to as “central bands”. Although we can hardly see gaps among the central bands on the scale of Fig. 2, we confirmed that even them are isolated from one another by finite gaps 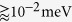 (see Fig. 2 in Supplemental Information D). Another thing we notice is that some neighboring bands, including the central ones, tend to converge toward M point (0.5, 0.5)*π*/(2*λ*). Regarding the dispersions, quite nice symmetry with respect to the energy 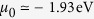 in the central bands also deserves close attention. Further analyses are needed in order to understand the secrets behind these interesting features.

Next, let us observe how the longitudinal conductivity *σ*_*xx*_ and AHC at *T* = 0 K, respectively equal to the function *e*^2^Σ_*xx*_(*ε*) and *e*^2^Σ_*xy*_(*ε*) in Eq. (2), depend on the band filling (Fermi energy) in Fig. 2(b,c) respectively. They are plotted in units of *e*^2^/*h*, and thus the latter value is the occupied-states-sum of Chern numbers itself (in the sense generalized to non-integers in metallic situation). The *σ*_*xx*_(*μ*)|_*T*=0_ has a large peak in the central bands region, as can be expected from the obviously large density of states there. The *σ*_*xx*_’s roughly symmetric variation with respect to *μ*_0_ (recognized when averaged over some broadened energy) could have been anticipated intuitively from the above-mentioned symmetric band structure.

The AHC, on the other hand, shows good anti-symmetric behavior with respect to *μ*_0_, which is quite understandable from the combined consideration of, again, the symmetric band structure and the assumption that each of the well isolated (other than the central) bands is analogous to a Landau level formed by external magnetic field which contributes AHC = 1 (*e*^2^/*h*) in the quantum Hall effect. The maximum absolute value of AHC is approximately 16, reached just below the central bands, while the second largest value of about 12 is located just above them (These maximums are smaller than the values 18 and 17 expected in an ideal case where no pair of bands overlaps at any energy, as is clear from Fig. 3 in Supplemental Information D). These behaviors result in the drastic change of AHC around the central bands as is prominent in Fig. 2(c). This very character has a strong effect on the TE properties of the system, as will be seen below.

### Filling-dependence of thermoelectric properties

We will proceed to our main subjects, the TE quantities of the system, especially focusing on their electron filling dependence, which we assume to be parametrized by the chemical potential *μ* within the rigid band approximation. In all what is reported hereafter, the room temperature *T* = 300 K is assumed.

**Figure 3:**
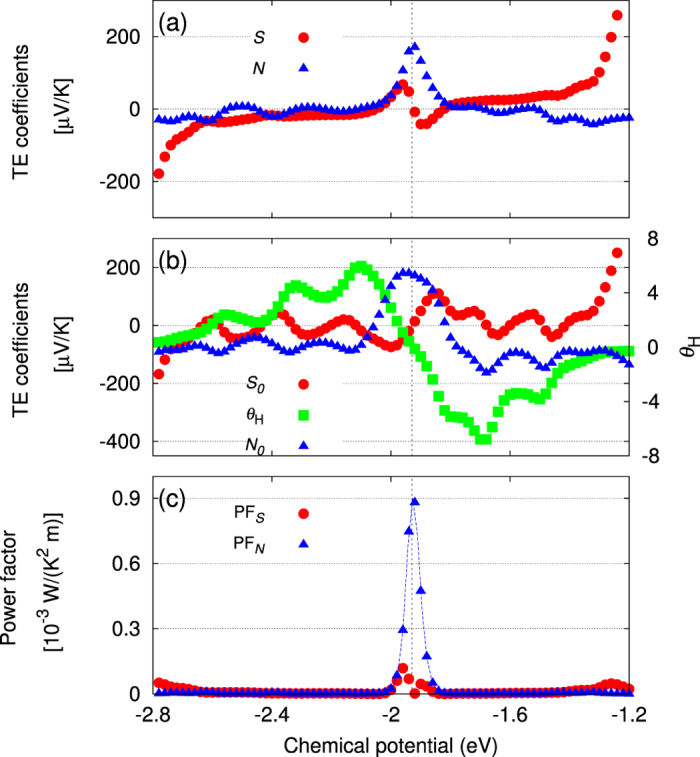
Chemical potential dependence of the thermoelectric quantities of 6 × 6 SkX at *T* = 300 K: (**a**) *S* and *N*, (**b**) *S*_0_, *N*_0_ (left axis) and *θ*_*H*_ (right axis) [see Eq. (1)], (**c**) Power factors corresponding to *S* and *N*. The blue dotted line for PF_*N*_ in (**c**) is drawn to guide the eye. The black dashed line indicates the *μ*_0_ mentioned in the main text.

In Fig. 3, (a) Seebeck *S* and Nernst *N* coefficients, (b) *S*_0_, *θ*_*H*_, and *N*_0_ [constituents of (a)], and (c) power factors associated with each of *S* and *N*, are shown, respectively.

As to the longitudinal *S*(*μ*), it is largely affected by the anomalous effect when *θ*_*H*_(*μ*) is finite (almost throughout the plotted *μ* range), except around the upper and lower edge, where 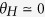. We recognize in Fig. 3(a,b) the following two points (i) and (ii) including their explanation based on what we have seen in Fig. 2: (i) Just below and above *μ*_0_, *S* shows the peaks (the higher one reaching ≈80 *μ*V/K) of the sign opposite to that of *S*_0_ despite rather small Hall angle *θ*_*H*_ there. This is thanks to the large *N*_0_ that satisfies |*θ*_*H*_ *N*_0_| > |*S*_0_|. (ii) Between the central and edge energy range, *S* is strongly suppressed compared to *S*_0_ due to large *θ*_*H*_.

On the transverse part *N*, what we notice in Fig. 3(a,b) and their interpretations are the following two points (i) and (ii): (i) Around *μ*_0_, it shows a large peak 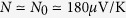. This is because of 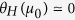 [see Eq. (1)] and large 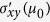 (the prime indicating the derivative with respect to *μ* at *T* = 0) as a result of the large anti-symmetry of *σ*_*xy*_ around *μ*_0_, which affects *N*_0_ approximately through the Mott’s relation [Eq. (3), later to be discussed quantitatively], which means 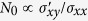. (ii) Away from *μ*_0_, *N* is strongly suppressed compared to *N*_0_ due to large *θ*_*H*_, similarly to the relation between *S* and *S*_0_. This is different from the situation where *N*_0_ is the far dominant contribution to *N*, as was previously reported^33^.

In view of the relation between *S* and *N*, it is instructive that large *N* appears thanks to the large asymmetry of *σ*_*xy*_, at the same filling where *S* diminishes due to the complete loss of asymmetry in *σ*_*xx*_, showing that *σ*_*xy*_ could be an additional freedom in seeking better thermoelectricity, when the material is properly designed.

Before concluding this subsection, we comment on our choice (*a*, *T*) = (3.3 Å, 300 K): This is just an example of suitable sets for finding large *N*_0_ in the following sense: If we obtain larger bandwidths, e.g. by compressing the lattice (making *a* smaller), while somehow keeping maximally the shape of Berry curvature, 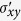 will be smaller, but thanks to the approximate relation 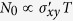 [see Eq. (3)], at a higher temperature we may well get *N*_0_ of the same magnitude as in the original system.

### Largest predicted thermoelectric voltages

In order to better capture quantitatively the above-mentioned connection between conductivities and TE coefficients that leads to large values of the latter, which are supposedly attractive for TE applications, let us check whether the largest values *S*_max_ and *N*_max_ seen in the central energy range in Fig. 3(a) can be roughly estimated via the well-known Mott’s formula, which says,


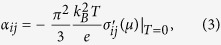


where *k*_*B*_ is the Boltzmann’s constant. We write the chemical potential values that respectively give *S*_max_ > 0 and *N*_max_ > 0 as *μ*_*S*_ and *μ*_*N*_, both of which are close to *μ*_0_ but slightly different from one another 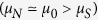. Finding that *S*_0_(*μ*_*S*_) ≈ −40 *μ*V/K, *θ*_*H*_(*μ*_*S*_) ≈ 1, *S* reduces to 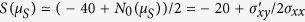. Similarly found is that 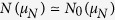 because of *S*_0_(*μ*_*N*_) ≈ 0 ≈ *θ*_*H*_(*μ*_*N*_). A rough estimation of 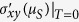 can be obtained by linearly-approximating the drastic drop of AHC Δσ_*xy*_ (*μ*) ≈ −28(*e*^2^/*h*)Ω^−1^ in the energy range Δ*μ* ≈ 0.13 eV in Fig. 2(c). This gives 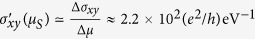. The other quantity we need is *σ*_*xx*_(*μ*_*S*_), which was found to be about 7.7(*e*^2^/*h*). Combining these, a dimensionless factor of 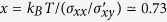 is found at *T* = 300 K 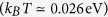. Finally our evaluation arrives at 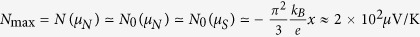 and therefore *S*_max_ = *S*(*μ*_*S*_) ≈ 1 × 10^2^ − 20 = 80 *μ*V/K. These values are in fairly good accordance with the integration-derived values, hence clarifying that *T* = 300 K is *low* enough (in comparison to the Fermi level) for the Mott’s formula to be valid at least for rough estimation.

At this point we compare the maximum value of *α*_*xy*_ ≈ 10^−8^ A/K corresponding to the above-estimated *N*_max_, with a value presumably around the upper bound within low-*T* approximation for the two-band Dirac-Zeeman (DZ) model we studied before^7^. The low-*T* approximated *α*_*xy*_ in Eq. (4) of our previous study^7^ can be rewritten as a 2D quantity 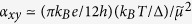, where Δ is the Zeeman gap and 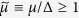 for the electron-doped case. Assuming rather small 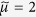, and choosing (*k*_*B*_*T*/Δ) = 0.3 to loosely satisfy the low-*T* criterion 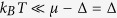, we obtain *α*_*xy*_ ≈ 10^−10^ A/K for the DZ model, which is by two orders of magnitude smaller than in the 6 × 6 SkX. Although some larger *α*_*xy*_ could be achieved beyond low-*T* range, still larger values in the SkX should be ascribed to the AHC more than ten times larger than that of the DZ model.

Finally, since 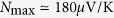 is a good enough value as a TE material, in order to further investigate the practical performance of the present system, we plot the power factor PF_*S*_ ≡ *σ*_*xx*_*S*^2^ and PF_*N*_ ≡ *σ*_*xx*_*N*^2^ in Fig. 3(c). Note that, for the evaluation of power factors, unlike in the discussions of TE quantities up to now, we need to know 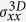. Therefore we assumed the SkX film thickness of 10 nm. The maximum of PF_*N*_ ≈ 10^−3^ W/(K^2^m) is comparable to the values of possible oxide TE candidates such as NaCo_2_O_4_ and ZnO^34^. Although the thermal conductivity *κ* was beyond the scope of this study, we just make a rough estimate here: Assuming the thermal conductivity *κ* of the order of 1 W/(Km) corresponding to good TE materials^34^, the present case realizes *Z*_*N*_*T* of the order of 0.3 at *T* = 300 K.

Please note, however, that the result is strongly dependent on the value of *τ* within our constant-*τ* approximation, as is clearly manifested in the relation *N*(*μ*_*N*_) ∝ *τ*^−1^. For example we obtain *N*(*μ*_*N*_) ≈ 20 *μ*V/K if we suppose *τ* = 1 ps, although the Nernst coefficient of this magnitude is still much larger than the so far reported values and is practically valuable^35^.

Observing the striking properties of SkX phase, we believe it should be an important task to reveal the mystery behind it.

## Additional Information

**How to cite this article**: Mizuta, Y. P. and Ishii, F. Large anomalous Nernst effect in a skyrmion crystal. *Sci. Rep.* **6**, 28076; doi: 10.1038/srep28076 (2016).

## Supplementary Material

Supplementary Information
