# Supplementary material for: Large anomalous Nernst effect in a skyrmion crystal
Source: Sci Rep. 2016 Jun 16;6:28076. doi: 10.1038/srep28076 (PMC4910103; doi:10.1038/srep28076)
Supplement: Supplementary Information [file srep28076-s1.pdf]

Supplemental Information:

Large anomalous Nernst effect in a skyrmion crystal

Yo Pierre Mizuta[1,*] and Fumiyuki Ishii[2]

[1]Graduate School of Natural Science and Technology, Kanazawa University, Kanazawa, 920-1192 JAPAN
[2]Faculty of Mathematics and Physics, Kanazawa University, Kanazawa, 920-1192 JAPAN
[1,*]mizuta@cphys.s.kanazawa-u.ac.jp
[2] ishii@cphys.s.kanazawa-u.ac.jp

A: Comment on the topological Hall contribution

We roughly estimate the magnitude of change in our result to be brought about by considering σ_{xy}^{T} omitted in the main body.

The expression for σ_{xy}^{T} in the Drude model treatment is,

$$\sigma_{xy}^{T} = \frac{\omega_s \tau}{1+(\omega_s \tau)^2}\sigma_{xx}, \tag{1}$$

where $\omega_s \equiv eB_{\text{spin}}/m_e$ with spin-scalar-chirality-induced field $B_{\text{spin}} \equiv (\hbar/2e)\hat{\mathbf{m}}\cdot(\partial_x\hat{\mathbf{m}}\times\partial_y\hat{\mathbf{m}})$ determined by the unit vector of the background spin texture $\hat{\mathbf{m}} \equiv \mathbf{m}/|\mathbf{m}|$ and electron mass m_e.

Substituting the present SkX form of $\hat{\mathbf{m}}(\mathbf{r})$, we obtain the spatially-inhomogeneous $B_{\text{spin}}(\mathbf{r}) = (\hbar/2e)(\pi/r\lambda)\sin\pi(1-r/\lambda)$, which gives a skyrmion size λ-independent flux of h/e per unit cell[1] . Approximating by taking its unit-cell averaged value $\overline{B}_{\text{spin}} = (h/e)/(\pi\lambda^2)$, which is roughly 10^3 T in our case of $\lambda \approx 1$nm, we obtain the frequency of $\overline{\omega_s} = e\overline{B}_{\text{spin}}/m_e \approx 2\times10^{14}\text{s}^{-1}$ and thus $\overline{\omega_s}\tau \approx 20$ for the scattering-relaxation time of $\tau = 0.1$ps assumed throughout this paper. Therefore, the contribution of $\sigma_{xy}^{T} \approx \sigma_{xx}/20$ adds less than 0.1 to θ_H and less than $0.1S_0$ to N_0, which is good reason to omit σ_{xy}^{T} in our case.

B: Mathematical expressions behind the form of conductivity tensors

The starting point to get the semiclassical formulae for conductivity tensors Eq.(5) is the expression for charge current $\mathbf{j}$ obtained by Xiao *et al.*[2] , whose two dimensional version reads,

$$\mathbf{j} = e\sum_n \int \frac{d\mathbf{k}}{(2\pi)^2} g_n(\mathbf{r},\mathbf{k})\mathbf{v}_{n\mathbf{k}} + e\nabla_{\mathbf{r}}\times k_{\text{B}}T(\mathbf{r})\sum_n \int \frac{d\mathbf{k}}{(2\pi)^2}\Omega_n(\mathbf{k})\log\left[1+\exp\left(-\frac{\varepsilon_{n\mathbf{k}}-\mu}{k_{\text{B}}T(\mathbf{r})}\right)\right], \tag{2}$$

where $e(<0)$, $g_n(\mathbf{r},\mathbf{k})$, $T(\mathbf{r})$, $\mathbf{v}_{n\mathbf{k}}$, $\varepsilon_{n\mathbf{k}}$, $\Omega_n(\mathbf{k})$, and μ stand for the electron's charge, distribution function, local temperature, (velocity, energy and $\mathbf{k}$-space Berry curvature) of an electron with wave number $\mathbf{k}$, and chemical potential, respectively, with the subscript index n put on each band-resolved quantity.

The second term is a correction that appears when spatial inhomogeneity [$T(\mathbf{r})$ in the present case] exists. Simplifying the second term following Xiao *et al.*[2] and substituting the set of equations of motion of a perturbed (by $\mathbf{E}$ field) Bloch electron

$$\mathbf{v}_{n\mathbf{k}} = \frac{1}{\hbar}\frac{\partial\varepsilon_{n\mathbf{k}}}{\partial\mathbf{k}} - \dot{\mathbf{k}}\times\Omega_n(\mathbf{k}), \;\; \dot{\mathbf{k}} = e\mathbf{E} \tag{3}$$

which was derived by Sundaram *et al.*[3] , for $\mathbf{v}_{n\mathbf{k}}$ and the form of distribution

$$g_n(\mathbf{r},\mathbf{k}) = \tau_{n\mathbf{k}}\mathbf{v}_{n\mathbf{k}}\cdot\left[e\mathbf{E}+(\varepsilon_{n\mathbf{k}}-\mu)\left(-\frac{\nabla T}{T}\right)\right]\left(-\frac{\partial f(\varepsilon_{n\mathbf{k}})}{\partial\varepsilon_{n\mathbf{k}}}\right), \tag{4}$$

obtained as the solution of Boltzmann transport equation within relaxation time ($\tau_{n\mathbf{k}}$) approximation, for $g_n(\mathbf{r},\mathbf{k})$, we obtain

the final expression:

$$
\begin{cases}
\sigma_{xx} = e^2\tau \sum_n \int \frac{d\mathbf{k}}{(2\pi)^2} \, [\mathbf{v}_{n\mathbf{k}}]_x^2 \left(-\frac{\partial f(\varepsilon_{n\mathbf{k}})}{\partial \varepsilon_{n\mathbf{k}}} \right), \\
\sigma_{xy} = -\frac{e^2}{\hbar} \sum_n \int \frac{d\mathbf{k}}{(2\pi)^2} \, [\Omega_n(\mathbf{k})]_z f(\varepsilon_{n\mathbf{k}}) = -\sigma_{yx}, \\
\alpha_{ij} = \frac{1}{e} \int d\varepsilon \sigma_{ij}(\varepsilon)|_{T=0} \frac{\varepsilon - \mu}{T} \left(-\frac{\partial f}{\partial \varepsilon} \right) \\
\qquad \text{for} \quad i = x \text{ or } y, \;\; j = x \text{ or } y,
\end{cases}
\tag{5}
$$

where $f(\varepsilon)$ is the Fermi-Dirac distribution, and we assumed a constant relaxation time ($\tau_{n\mathbf{k}} = \tau$).

The expressions in Eq.(5), though looking different, are equivalent to the ones presented around Eq.(2) of the main body.

C: Skyrmion size dependence

The maximum value of Nernst coefficient N at $T = 300$K found in the space of chemical potential μ is plotted as a function of the skyrmion size n^2 (number of sites forming a single skyrmion) in Fig.1. We clearly see a monotonic grow of $N_{\max}$ that

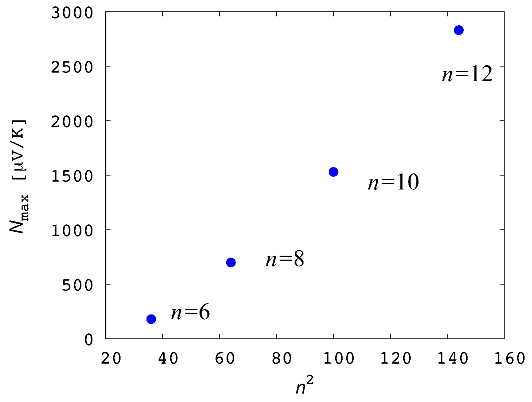

Figure 1. Variation of the maximum N in the space of μ as the skyrmion size (n^2) grows.

we can interpret as a direct consequence of larger maximum values of the anomalous Hall conductivity (AHC) for larger n (in consistent with the statement by Hamamoto *et al.*[1]) , which suddenly drop in almost n-independent energy width (i.e. width barely changed from the one for $n = 6$ case seen in Fig.2 of the main body). The behavior of roughly $N_{\max} \propto n^2$ is understood from the contribution of AHC=1(e^2/h) from each of many bands as is mentioned in the main body. Although this trend seems very promising, we need to be careful of the problem of scattering, as disorder of the same extent has *more* serious effect on the electron conduction over *larger* unit cells.

D: Berry curvature and band-resolved Chern number

We have performed first-principles calculation of Berry curvature $\Omega(\mathbf{k})$ and Chern number C_n for each band (n-th from the bottom) also directly from the Bloch wave functions by using discretized formula.[4,5] The former (and the corresponding band dispersion) and the latter are plotted in Fig.2 and Fig.3, respectively. The results are consistent with those of *Wannier90* reported in the main body, evaluated via Wannier functions constructed from the Bloch wave functions. What is particularly interesting is that the 21th band indicated in red in the upper panel of Fig.2 has an extremely large Chern number of $C_{21} = 27$ as shown in Fig.3. The evaluation of $\Omega(\mathbf{k})$ on this band (lower panel of Fig.2) shows sharp peaks at every position of avoided crossing with the adjacent band, which look larger in magnitude for smaller gaps (An enlarged plot of the seemingly smallest gap where $\Omega(\mathbf{k})$ takes the maximum is displayed inside Fig.2). This behavior is a clear manifestation of the character of Kubo-formula accounting for the Berry curvature as its partial contribution. Most of the bands, i.e. except three (19th-21th), have $C_n = 1$, somewhat reminiscent of Landau levels in the quantum Hall effect. The Chern number of each band should manifest itself as the number of chiral edge states which additionally appear in the gap just above that band (bulk-edge correspondence) as explicitly exemplified by Hamamoto *et al.*[1] It is of great interest and also a future task to reveal the origin (key factor)

of such a very inhomogeneous distribution of Chern numbers, which realizes the excellent thermoelectric performance we reported in the main body.

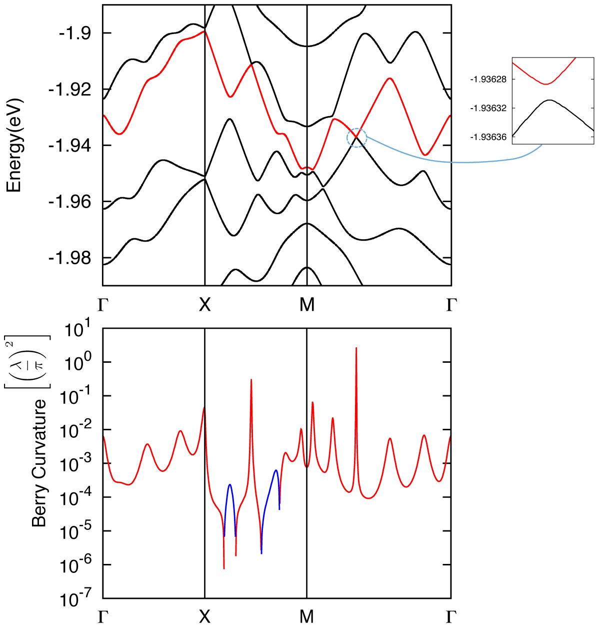

Figure 2. (Top) Band dispersion of central bands and (Bottom) Berry curvature on 21th (from the lowest) band (red line in the top panel) along the path Γ-X-M-Γ. The latter is in logarithmic scale and the red (blue) part indicates its positive (negative) value. The Berry curvature is in unit of $(\lambda/\pi)^2$, where λ is half the value of the lattice constant.

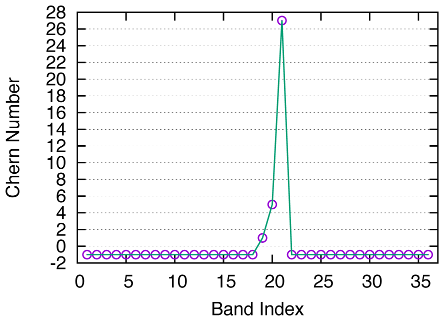

Figure 3. The band index dependence of Chern number.

E: Validity of our model and future tasks

While showing noticeable properties as presented in this paper, the model we studied has several limitations in its validity in comparison with real nature, of which we categorize into four (though interrelated): (1) Orbital species, (2) Stability of target magnetic states, (3) Electron filling dependence, and (4) Carrier scattering effects. Here are their details: (1) Our model adopted the hydrogen atom, which has only one electron per site with dominantly s-orbital character, while real SkX systems usually have large contributions from multiple d-orbitals with non-negligible effect of spin-orbit coupling. In this regard, we have confirmed similar behavior of Chern numbers in p- and d-orbital cases with spin-orbit coupling as our preliminary computations. (2) Our model realized its SkX state thanks to the artificial constraint on its spins, while real SkX are resulting from delicate balance among complicated magnetic interactions. Therefore the next step will be to investigate the system energetics. (3) Here we assumed rigid bands, i.e. the chemical potential shift meant addition(subtraction) of carriers into(from) frozen bands, while μ in real materials is determined self-consistently, whose effects we can study by introducing additional charge in our present first-principles approach, or by resorting to more sophisticated alloying simulations based on coherent potential approximation. (4) A constant value of carrier mean-free-time τ was arbitrarily chosen here, while in reality it is determined from the system details including its energy- and temperature- dependencies. Such effects can be taken into account within some approximations, using first-principles electronic states.

References

1. Hamamoto, K., Ezawa, M. & Nagaosa, N. Quantized topological Hall effect in skyrmion crystal. *Phys. Rev. B* **92**, 115417 (2015).
2. Xiao, D., Yao, Y., Fang, Z. & Niu, Q. Berry-phase effect in anomalous thermoelectric transport. *Phys. Rev. Lett.* **97**, 026603 (2006).
3. Sundaram, G. & Niu, Q. Wave-packet dynamics in slowly perturbed crystals: Gradient corrections and Berry-phase effects. *Phys. Rev. B* **59**, 14915 (1999).
4. Fukui, T., Hatsugai, Y. & Suzuki, H. Chern Numbers in Discretized Brillouin Zone: Efficient Method of Computing (Spin) Hall Conductances. *J. Phys. Soc. Jpn.* **74**, 1674 (2005).
5. Feng, W., Wen, J., Zhou, J., Xiao, D. & Yao, Y. First-principles calculation of Z_2 topological invariants within the FP-LAPW formalism. *Comput. Phys. Commun.* **183**, 1849 (2012).
